# Risk Stratification of Postpartum Patients with Hypertensive Disorders of Pregnancy to Telehealth Follow-Up: A Quality Improvement Project

**DOI:** 10.1055/a-2788-9872

**Published:** 2026-02-02

**Authors:** Ariana M. Banuelos, Kalin Ellison, Weirui Xiao, Patricia Chavez, Diana S. Wolfe, Anna E. Bortnick, Kavita Vani

**Affiliations:** 1 Department of Obstetrics, Gynecology and Women's Health, Division of Maternal Fetal Medicine, Montefiore Medical Center and Albert Einstein College of Medicine, Bronx, New York, United States; 2 Albert Einstein College of Medicine, Bronx, New York, United States; 3 Department of Medicine, Division of Cardiology, Montefiore Medical Center and Albert Einstein College of Medicine, Bronx, New York, United States; 4 Maternal Fetal Medicine-Cardiology Joint Program, Montefiore Medical Center and Albert Einstein College of Medicine, Bronx, New York, United States; 5 Department of Medicine, Division of Geriatrics, Montefiore Medical Center and Albert Einstein College of Medicine, Bronx, New York, United States

**Keywords:** hypertension, postpartum, follow-up, quality improvement, telehealth

## Abstract

**Background:**

Guidelines recommend a 3-day follow-up for severe hypertensive disorders of pregnancy (HDP) and a 7- to 10-day follow-up for nonsevere HDP, but implementation varies.

**Objective:**

To improve adherence to guideline-recommended postpartum follow-up by targeting provider discharge recommendations. A risk stratification tool incorporating HDP severity, maternal symptoms, and discharge blood pressure guided providers to recommend telehealth follow-up at 3, 5, or 7 days. We aimed to maintain 3-day recommendations for severe HDP and reduce unnecessary 3-day recommendations for nonsevere HDP.

**Study Design:**

This quality improvement project was conducted at a single urban academic institution. The risk stratification tool was integrated into discharge workflows, and demographic, clinical, and telehealth follow-up data for pre- and post-intervention cohorts were abstracted from the electronic medical record and compared using summary statistics and bivariate analyses.

**Results:**

Cohorts were similar at baseline. After implementation, all patients with severe HDP continued to receive 3-day follow-up instructions. Among patients with nonsevere HDP patients, 3-day recommendations decreased from 100 to 31.1% ( *p* < 0.001), with a corresponding decrease in 3-day telehealth scheduling. Scheduling for severe HDP did not improve.

**Conclusion:**

The tool improved guideline-aligned provider recommendations for postpartum HDP. Scheduling changes were limited, suggesting future efforts should target workflow and system processes.

## Introduction

Hypertensive disorders of pregnancy (HDP) complicate approximately 16% of deliveries in the United States and are associated with increased risks of maternal morbidity, such as cardiac arrest and stroke, maternal mortality, and long-term cardiovascular disease, including chronic hypertension, coronary artery disease, and heart failure. 1 2 These risks disproportionately affect individuals with lower socioeconomic status and historically marginalized populations, with maternal mortality rates up to three times higher among Black compared to White patients. 3

HDP encompasses a spectrum of diagnoses of varying severity, including chronic hypertension, gestational hypertension, preeclampsia, and eclampsia, with increasing severity associated with greater maternal morbidity and mortality. 4 5 6 The American College of Obstetricians and Gynecologists (ACOG) and the Society for Maternal Fetal Medicine (SMFM) define severe HDP as preeclampsia or chronic hypertension with superimposed preeclampsia with severe features, eclampsia and hemolysis, elevated liver enzymes, low platelets (HELLP) syndrome, and nonsevere HDP as chronic hypertension, gestational hypertension, and preeclampsia without severe features. 4 5 Because preventable complications such as exacerbation of or new-onset severe hypertension often arise within the first 1 to 2 weeks postpartum, ACOG and SMFM recommend short-interval postpartum follow-up for all patients with HDP, specifying follow-up within 3 days for those with severe HDP and within 7 to 10 days for those with nonsevere HDP. 2 7 Implementation of these guidelines is institution-specific, and a risk-stratified approach to follow patients with HDP after delivery discharge has not been previously investigated.

Telehealth has been associated with improved blood pressure monitoring within the first 10 days postpartum, 8 9 increased adherence amongst Black patients, 10 and reduced hospital readmissions. 9 Accordingly, our institution employs telehealth for short-interval postpartum follow-up of patients with HDP. However, in our high-risk patient population, in which the prevalence of HDP is approximately 25%, obstetric providers have historically recommended follow-up within 3 days of discharge for all patients with HDP, regardless of severity. This practice exceeded clinic capacity, resulting in only 11% of all HDP patients being scheduled within the requested 3 days, disproportionately limiting timely follow-up for those with severe disease who most required early evaluation.

**Fig. 1:**
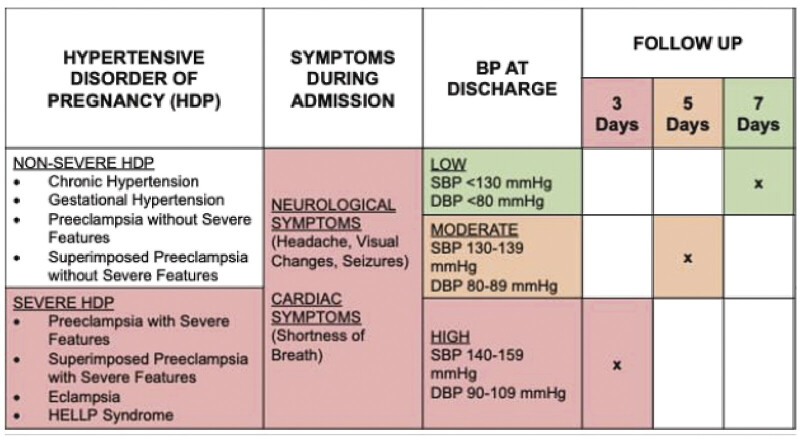
Risk stratification tool for scheduling follow-up of patients with hypertensive disorders of pregnancy.

To address this gap, we conducted a quality improvement project focused on provider behavior at discharge. We developed and implemented a risk stratification tool ( Fig. 1 ) to guide follow-up recommendations by disease severity and other clinical factors. The primary aim was to maintain provider requests for 3-day follow-up among patients with severe HDP by redistributing provider requests to 3-, 5-, or 7-day follow-up for patients with nonsevere HDP. A secondary aim was to evaluate provider adoption of the algorithm and whether this change improved the actual scheduling of 3-day telehealth visits for patients with severe HDP.

## Materials and Methods

### Context

The Jack D. Weiler Hospital of the Montefiore Health System serves a predominantly Black and Hispanic and publicly insured patient population in the Bronx, New York. At our institution, postpartum telehealth follow-up is determined by the inpatient obstetric providers (residents, attendings, and physician assistants) during the immediate postpartum period. Patients with HDP are prescribed a blood pressure cuff at discharge and instructed on its appropriate use. The follow-up timing is listed in the patient's discharge summary by the provider and routed to the schedulers for appointments to be made. Prior to the intervention described, it was standard practice for providers to request a blood pressure telehealth appointment for 3 days after discharge for all patients with HDP, regardless of disease severity.

Based on the Plan-Do-Study-Act (PDSA) framework for Quality Improvement, 11 a multidisciplinary group at our institution including maternal–fetal medicine and cardiology physicians completed a review of the current discharge practice of patients with HDP, then developed a risk stratification tool that included 3 factors: HDP diagnosis, blood pressure at the time of discharge, and any symptoms concerning for severe preeclampsia experienced during the delivery admission ( Fig. 1 ) to determine if a patient should be recommended for a telehealth visit for blood pressure check in 3, 5, or 7 days postdischarge. According to the tool, those who had a diagnosis of severe HDP, as described above, and/or a higher blood pressure at discharge, and/or symptoms during admission would be instructed to follow up at a shorter interval compared to those without severe hypertension, normal blood pressure at discharge, and without severe symptoms during admission. The study was approved by the Albert Einstein College of Medicine Institutional Review Board. This study was granted a waiver of informed consent by the Institutional Review Board, given that this was a quality improvement project that involved retrospective analysis of de-identified patient information.

### Intervention(s)

Intervention #1: Weekly education sessions were conducted after postpartum interdisciplinary team (IDT) rounds between July and August 2023. Postpartum IDT rounds are a meeting between nurses, physicians, advanced practice providers, hospital administrators, and social workers where clinical care is coordinated and discharge is planned. The education sessions were conducted by a member of our team with the obstetrical providers responsible for determining discharge follow-up for postpartum patients. The education sessions included standardized background information and introduction of the risk stratification tool for follow-up appointments ( Fig. 1 ). The educational session was held weekly as the attending and intern who manage the postpartum patients rotate weekly. In addition to these in-person teaching sessions, the risk stratification tool was printed and accessible near postpartum provider workstations to reinforce use of the tool for follow-up appointment requests.

Intervention #2: An educational session was held with the intern class of the Obstetrics and Gynecology residency program on August 29, 2023, as interns primarily manage the postpartum service. The content of this session was the same as described in Intervention #1.

Intervention #3. An educational session was held during an Obstetrics and Gynecology Generalist Division meeting on September 28, 2023. The education session contained the same content as Intervention #1 but specifically targeted 28 attendings of the Generalist Division who rotate weekly as the postpartum attending.

### Measures

Because provider behavior change was expected to occur gradually rather than at a single time point, we defined the post-intervention period as July 2023 to February 2024 to capture the full implementation and adoption phase, including the ramp-up period. Delivery discharges between July 2023 and February 2024 were reviewed in discrete waves of time before, during, and after the interventions to identify patients with a clinical diagnosis of severe and nonsevere HDP as defined by ACOG. 4 5 A pre-intervention cohort of patients with HDP discharged in February 2023 was also identified for comparison. De-identified data were abstracted from the electronic medical record by nonblinded medical student and physician reviewers.

For identified patients with HDP pre- and post-intervention, deidentified data was collected and entered into the Research Electronic Data Capture (REDCap) database including demographics, delivery details, HDP diagnosis, blood pressure at discharge, symptoms concerning for severe disease during hospitalization (headache, visual symptoms, chest pain, and/or shortness of breath), provider instructions for telehealth follow-up timing within 3, 5 or 7 days, scheduling and attendance of a telehealth appointment, and readmission for HDP after discharge.

### Analysis

Demographics, HDP characteristics, and telehealth visit data were compared between the pre- and post-intervention cohorts. Summary statistics were used to describe the distribution of recommended discharge follow-up in the pre- and post-intervention time periods per the risk stratification tool and actual by obstetric provider. Categorical variables were compared using the chi-square test, and continuous variables were compared using the *t* -test and Mann–Whitney U test, as appropriate. Statistical significance was defined as *p* < 0.05, and analyses were performed in Stata v. 17.0 (College Station, Texas, United States).

## Results

### Baseline Characteristics

The pre- and post-intervention cohorts, which included all patients with any HDP diagnosis as described above, were similar with respect to age, race-ethnicity, mode of delivery, and HDP characteristics ( Table 1 ). The mean age was 31 ± 6 years old. Most patients identified as non-White, of whom 29.8% ( *n* = 168) identified as Black and 54.5% ( *n* = 307) identified as Hispanic. Cesarean birth was performed in 43.3% ( *n* = 224) of patients. The proportion of severe HDP diagnoses, including preeclampsia with severe features, chronic hypertension with superimposed preeclampsia with severe features, eclampsia, and HELLP syndrome, was the same between both cohorts (25.6% vs. 30.7%, *p* = 0.26). Overall, the mean systolic blood pressure at discharge was 126 ± 10 mmHg, the mean diastolic blood pressure at discharge was 80 ± 8 mmHg, and 28.6% ( *n* = 160) were prescribed antihypertensive medication at discharge.

**Table 1:** Baseline characteristics of patients with hypertensive disorders of pregnancy pre- and post-intervention with a risk-stratification tool for follow-up scheduling

	All ( *n* = 563)	pre-intervention ( *n* = 133)	post-intervention ( *n* = 430)	*p* -Value
Age, y (mean ± SD)	30.7 ± 6.2	30.1 ± 6.5	30.6 ± 6.1	0.39
Race ( *n* ,%) White Black Asian Hawaiian/PI Unknown Other	24 (4.3) 168 (29.8) 12 (2.1) 1 (0.2) 75 (13.3) 283 (50.3)	4 (3.0) 29 (21.8) 3 (2.3) 0 (0) 21 (15.8) 76 (57.1)	20 (4.7) 139 (32.3) 9 (2.1) 1 (0.2) 54 (12.6) 207 (48.1)	0.21
Ethnicity ( *n* ,%) Hispanic Non-Hispanic	256 (45.5) 307 (54.5)	69 (51.9) 64 (48.1)	187 (43.5) 243 (56.5)	0.09
Mode of delivery ( *n* ,%) Vaginal Cesarean Operative vaginal	309 (54.9) 244 (43.3) 10 (1.8)	74 (55.6) 56 (42.1) 3 (2.3)	235 (54.7) 188 (43.7) 7 (1.6)	0.86
Any symptoms during admission a ( *n* , %) Yes No	75 (13.3) 488 (86.7)	15 (11.3) 118 (88.7)	60 (14.0) 370 (86.0)	0.43
Length of admission, d (median, IQR)	4 (3–5)	4 (3–5)	4 (3–5)	0.64
Diagnosis of severe HDP b ( *n* ,%) Yes No	166 (29.5) 397 (70.5)	34 (25.6) 99 (74.4)	132 (30.7) 298 (69.3)	0.26
Systolic BP at discharge, mmHg (mean ± SD)	126 ± 10	126 ± 10	126 ± 10	0.23
Diastolic BP at discharge, mmHg (mean ± SD)	80 ± 8	81 ± 8	79 ± 8	0.09
Antihypertensive prescribed at discharge ( *n* , %) Yes No	160 (28.6) 400 (71.4)	31 (23.8) 102 (77.3)	129 (30.2) 298 (69.8)	0.12

Abbreviations: BP, blood pressure; HDP, hypertensive disorder of pregnancy; HELLP, hemolysis, elevated liver tests, low platelets; PI, Pacific Islander.

a Presence of headaches, visual symptoms, shortness of breath, seizures

b Includes preeclampsia with severe features, superimposed preeclampsia with severe features, eclampsia, and HELLP syndrome

### Implementation/Use of the Risk Stratification Tool

**Fig. 2:**
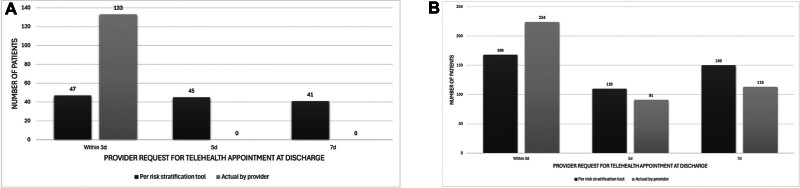
( **A** ) pre-intervention timing of follow-up for patients with hypertensive disorders of pregnancy according to the risk stratification tool (black) versus actual practice (gray). ( **B** ) post-intervention timing of follow-up for patients with hypertensive disorders of pregnancy according to the risk stratification tool (black) versus actual practice (gray).

During the pre-intervention time frame, 100% ( *n* = 133) of all patients with HDP, severe and nonsevere, were recommended to follow up within 3 days after discharge per institutional practice. As a baseline, the risk stratification tool was retrospectively applied to this pre-intervention cohort and resulted in the following distribution of follow-up timing recommendations: 35.3% ( *n* = 47) within 3 days, 33.8% ( *n* = 45) within 5 days, and 30.8% ( *n* = 41) within 7 days ( Fig. 2A ). These baseline pre-intervention cohort recommendations were concordant with the risk stratification tool for 35.3% of the patients. During the post-intervention time frame ( *n* = 430), the risk stratification tool was implemented by the discharging obstetric providers, achieving a time distribution of follow-up recommendations: 52.5% ( *n* = 226) within 3 days, 21.2% ( *n* = 91) within 5 days, and 26.3% ( *n* = 113) within 7 days. The concordance between follow-up telehealth visit recommendations by the discharging obstetric providers and the risk stratification tool increased with its implementation (35.3% pre-intervention vs. 61.4% post-intervention, *p* < 0.01). Fig. 2B demonstrates the post-intervention timing of follow-up for patients with HDP, per provider instructions and per risk stratification tool.

### Primary Measure

For patients with severe HDP, 100% ( *n* = 132) of patients were instructed to follow up within 3 days of discharge during the post-intervention time frame, consistent with society recommendations and local practice.

### Secondary Measures

**Fig. 3:**
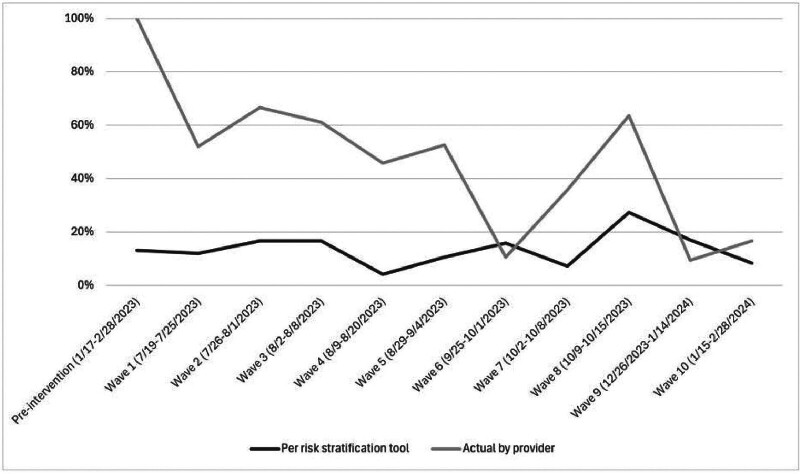
Change in 3-day follow-up instructions for individuals with nonsevere hypertensive disorders of pregnancy. (black = classification by risk stratification tool, gray = classification by obstetric provider)

There was a decrease in the proportion of telehealth visit recommendations by obstetric providers for patients with nonsevere HDP within 3 days of discharge, from 100% ( *n* = 99) in the pre-intervention cohort to 31.1% ( *n* = 92) in the post-intervention cohort ( *p* < 0.001). Fig. 3 demonstrates the postdischarge follow-up instructions for patients with nonsevere HDP within 3 days, per provider instructions and per risk stratification tool, during the time of the intervention.

For all HDP patients, discharge follow-up instructions were routed to the schedulers for scheduling of the postpartum appointments. No changes were made to the process by which appointments were scheduled during the post-intervention time frame. There was no significant difference in the proportion of patients scheduled for any telehealth visit during the pre- or post-intervention time frames among nonsevere (74.8% vs. 81.9%, *p* = 0.12) and severe (79.4% vs. 82.6%, *p* = 0.67) HDP patients. The proportion of severe HDP patients scheduled for a telehealth visit within 3 days of discharge was similar between pre- and post-intervention cohorts (56.0% vs. 44.0%, *p* = 0.28). There was a decrease in the proportion of nonsevere HDP patients scheduled for a telehealth visit within 3 days of discharge (39.2% pre vs. 12.7% post, *p* < 0.01). Among patients scheduled, there was no difference in the attendance rate for telehealth visits in pre- or post-intervention time frames for nonsevere (83.8% vs. 82.4%, *p* = 0.78) or severe (77.8% vs. 84.4%, *p* = 0.41) HDP.

Lastly, there was no difference in the postpartum readmission rate for preeclampsia in the pre- and post-intervention groups (6.8% vs. 6.8%, *p* = 0.99).

## Discussion

With this quality improvement initiative, we created a risk stratification tool to guide provider discharge recommendations for postpartum follow-up among patients with HDP in alignment with ACOG guidelines. The tool was designed to ensure that patients with severe HDP continued to be prioritized for follow-up within 3 days of discharge, while allowing patients with nonsevere HDP to be scheduled at later intervals when clinically appropriate. We achieved our primary aim: provider recommendations for 3-day follow-up among patients with severe HDP were maintained, while recommendations for nonsevere HDP shifted away from universal 3-day follow-up and decreased to 31.1%. This redistribution reflects a meaningful change in provider behavior toward guideline-concordant, severity-based follow-up decision making.

While ACOG and SMFM have clear guidelines that recommend expedited follow-up for patients with HDP depending on its severity, there is limited guidance on how to operationalize these recommendations in high-volume clinical settings. 3 4 By introducing our risk stratification tool and integrating it into routine discharge workflows with targeted provider education, we demonstrate a feasible strategy to implement guideline-aligned, risk-stratified postpartum follow-up.

The concept of risk stratification at hospital discharge is understudied within obstetrics and gynecology. However, its use in other specialties demonstrates that risk stratification tools at the time of hospital discharge, such as the LACE index, which incorporates length of stay, acuity of admission, comorbidity index, and history of emergency room visit, can be useful to prevent re-hospitalization and improve outcomes. 12 13 Efforts to classify patients into high and low risk strata by using a multifactorial tool at the time of discharge may improve resource utilization and provider recommendations for timing of follow-up. 14

This initiative specifically targeted provider behavior at the time of discharge rather than downstream clinic operations. Accordingly, while provider recommendations shifted as intended, there was no parallel increase in the proportion of patients with severe HDP who were actually scheduled within 3 days. Scheduling workflows involves multiple additional steps and personnel who were not included in this PDSA cycle. It is possible that the redistribution of follow-up recommendations was interpreted by schedulers as increased flexibility in appointment timing, or that existing telehealth capacity constraints limited scheduling changes despite new recommendations. The lack of improvement in scheduling for severe HDP patients indicates a gap between provider recommendations and operational workflow. While readmissions did not increase in this sample, timely follow-up for severe HDP remains an important safety priority, and this represents a key area for future system-level intervention.

By incorporating maternal symptoms and discharge blood pressures in addition to HDP severity, the proposed risk stratification tool offers a more specific approach to identifying patients who may require closer surveillance. For those not yet meeting criteria for a diagnosis of severe HDP, maternal symptoms may predict impending complications of preeclampsia, 15 and discharge blood pressure may predict need for initiation or titration of antihypertensive medication to prevent spikes in blood pressure in the immediate postpartum period. 16 The clinical factors included in the tool may help identify patients at risk for readmission and enhance our ability to more promptly identify patients at risk of complications to modify treatment. 16 17 This allows for a more conservative yet individualized follow-up plan, particularly for patients with nonsevere HDP.

This initiative was a multidisciplinary effort that utilized available knowledge to develop a nuanced strategy while adhering to ACOG and SMFM guidelines for timely postpartum HDP follow-up after discharge in a racially diverse and socioeconomically disadvantaged patient population. An additional strength was the use of a detailed chart review of all discharged obstetric patients from our hospital to accurately identify and track postpartum follow-ups for all patients with a HDP diagnosis in both pre- and post-intervention groups. Lastly, the analysis was performed according to disease severity only (i.e., without the additional factors in the proposed tool), to adhere to established classifications by major obstetric societies.

The most significant limitation of this initiative, as described above, is that the intervention targeted provider recommendations only and did not address the downstream scheduling workflow required to ensure that recommended follow-up telehealth visits occurred at the intended time. Another limitation is that the education intervention did not reach all discharging providers. Our intervention targeted weekday teams. Subspecialty attendings, fellows, and senior residents who round and provide discharge instructions for postpartum patients on weekends were not included in the education cycle. Additionally, some discharging providers may have relied on clinical judgment over the risk stratification tool in determining postpartum follow-up for patients with HDP.

## Conclusion

To our knowledge, this quality-improvement initiative is the first study to risk-stratify the timing of immediate postpartum follow-up for patients with HDP after delivery hospitalization using clinical factors in addition to the severity of diagnosis. This approach allowed provider recommendations to be aligned with guidelines while prioritizing high-risk patients for earlier evaluation. The multifactorial assessment of the patients' complexity led to a distribution in the recommended postpartum follow-up for patients with HDP between 3 and 7 days after discharge. Future quality improvement goals for HDP postpartum follow-up at our institution will focus on aligning scheduling workflows so that recommended follow-up intervals translate into completed telehealth visits. Additionally, further studies are needed to elucidate the clinical factors most predictive of postpartum readmission, which can help refine this tool for future generalized use.
